# Automatic Parameter Tuning for Adaptive Thresholding in Fruit Detection

**DOI:** 10.3390/s19092130

**Published:** 2019-05-08

**Authors:** Elie Zemmour, Polina Kurtser, Yael Edan

**Affiliations:** Department of Industrial Engineering and Management, Ben-Gurion University of the Negev, Beer-Sheva 8410501, Israel; kurtser@post.bgu.ac.il (P.K.); yael@bgu.ac.il (Y.E.)

**Keywords:** adaptive thresholding, fruit detection, parameter tuning

## Abstract

This paper presents an automatic parameter tuning procedure specially developed for a dynamic adaptive thresholding algorithm for fruit detection. One of the major algorithm strengths is its high detection performances using a small set of training images. The algorithm enables robust detection in highly-variable lighting conditions. The image is dynamically split into variably-sized regions, where each region has approximately homogeneous lighting conditions. Nine thresholds were selected to accommodate three different illumination levels for three different dimensions in four color spaces: RGB, HSI, LAB, and NDI. Each color space uses a different method to represent a pixel in an image: RGB (Red, Green, Blue), HSI (Hue, Saturation, Intensity), LAB (Lightness, Green to Red and Blue to Yellow) and NDI (Normalized Difference Index, which represents the normal difference between the RGB color dimensions). The thresholds were selected by quantifying the required relation between the true positive rate and false positive rate. A tuning process was developed to determine the best fit values of the algorithm parameters to enable easy adaption to different kinds of fruits (shapes, colors) and environments (illumination conditions). Extensive analyses were conducted on three different databases acquired in natural growing conditions: red apples (nine images with 113 apples), green grape clusters (129 images with 1078 grape clusters), and yellow peppers (30 images with 73 peppers). These databases are provided as part of this paper for future developments. The algorithm was evaluated using cross-validation with 70% images for training and 30% images for testing. The algorithm successfully detected apples and peppers in variable lighting conditions resulting with an F-score of 93.17% and 99.31% respectively. Results show the importance of the tuning process for the generalization of the algorithm to different kinds of fruits and environments. In addition, this research revealed the importance of evaluating different color spaces since for each kind of fruit, a different color space might be superior over the others. The LAB color space is most robust to noise. The algorithm is robust to changes in the threshold learned by the training process and to noise effects in images.

## 1. Introduction

Fruit detection is important in many agricultural tasks such as yield monitoring [1,2,3,4,5,6,7,8], phenotyping [9,10,11], precision agriculture operations (e.g., spraying [12] and thinning [13,14,15]), and robotic harvesting [16,17,18]. Despite intensive research conducted in identifying fruits, implementing a real-time vision system remains a complex task [17,18]. Features like shape, texture, and location are subject to high variability in the agricultural domain [18]. Moreover, fruits grow in unstructured environments with highly-variable lighting conditions [19,20] and obstructions [21] that influence detection performance. Color and texture are fundamental characteristics of natural images and play an important role in visual perception [22].

Nevertheless, despite the challenges, several algorithms have been developed with impressive detection rates of over 90–95%. However, these detection rates were achieved only for specific fruit (apples, oranges, and mangoes) [13,23,24]. These crops are known for their high ratio of fruits per image allowing easier acquisition of large quantities of data. Other crops such as sweet peppers and rock melons [23] with a lower fruit-to-image ratio yield lower results of 85–90% [19,23], even with the employment of cutting edge techniques such as deep learning [25]. Additionally, the crops with high detection rates with these results are very distinct from their background in terms of color, a central feature for a color-based-only detection. The only recent research made to detect green crops is on weed detection [26,27], but these green crops are held against a brown background. In grape clusters’ detection [28], a rate of about 90% accuracy was achieved. Some work in the development of cucumber harvesters [29] has been done, but the success rate of harvesting was not distinguished from detection success rates and therefore cannot be reported.

In this research, we focus on the detection of three different types of challenging crops: red apples (a high ratio of fruits per image; however, we used a very small dataset http://icvl.cs.bgu.ac.il/lab_projects/agrovision/DB/Sweeper05/#/scene), green grapes (“green-on-green” dataset [28]), and yellow sweet peppers (a low fruit-to-image ratio http://icvl.cs.bgu.ac.il/lab_projects/agrovision/DB/Sweeper06/#/scene).

The adaptive thresholding algorithm presented in this paper is based on previous work [19] that was developed for sweet peppers’ detection for a robotic harvester. A set of three thresholds was determined for each region of the image according to its lighting setting. Preliminary results of the same algorithm for an apple detection problem have been previously presented [30].

The current paper advances previous research [30] with several new contributions: (1) a new parameter tuning procedure developed to best-fit the parameters to the specific database; (2) the application and evaluation of the adaptive thresholding algorithm for different color spaces; (3) application of the algorithm to different types of fruits along with intensive evaluation and sensitivity analyses; (4) comparing the contribution of the new developments (Items 1–2) to previous developments.

## 2. Literature Review

### 2.1. Detection Algorithms in Agriculture

While this paper does not aim to be a review paper, in addition to the many recent reviews (e.g., [16,18,25]), a summary of previous results helps place the outcomes of this paper into context (Table 1).

As can be seen in the table, most algorithms focus on pixel-based detection (e.g., segmentation). This is indeed a common method in fruit detection (e.g., [31,32,33,34]). Many segmentation algorithms have been developed [35] including: K-means [36], mean shift analysis [37], Artificial Neural Networks (ANN) [38], Support Vector Machines (SVM) [39], deep learning [25], and several others.

A common challenge facing agriculture detection research is the lack of data [20], due to the harsh conditions for image acquisition and the tedious related ground truth annotation [40]. Current advanced algorithms (e.g., deep learning) require collecting many data. Therefore, to date, the best detection results are provided for crops with high fruit-to-image ratios (e.g., apples, oranges, and mangoes) and fruits that grow in high density, and hence, each image provides many data. Some research [26,41] aimed to cope with the need for large quantities of highly-variable data by pre-training a network on either non-agricultural open access data [26] or by generating synthetic data [41]. Both methods have shown promising results.

**Figure 1:**
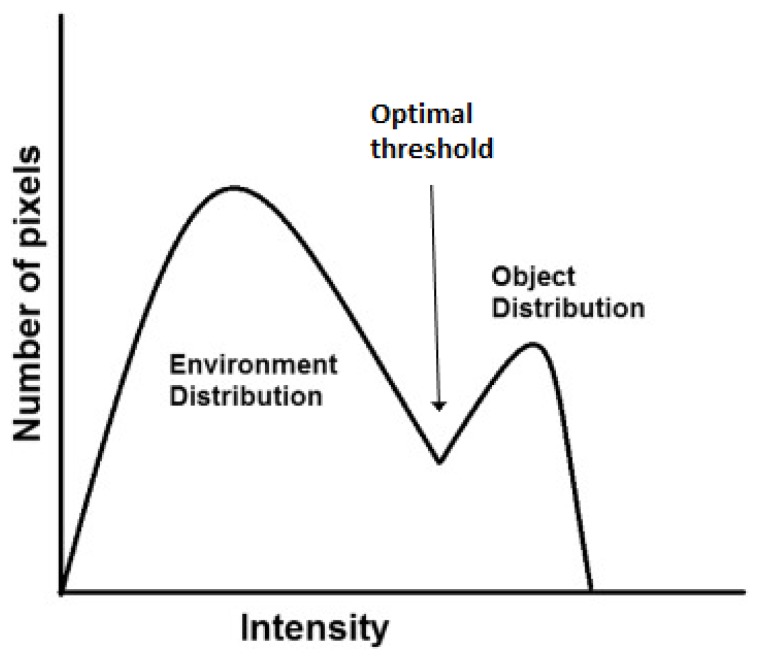
Optimal threshold in the bimodal histogram.

In this paper, we present an alternative direction, focusing on the development of algorithms based on smaller datasets. This research focuses on segmenting objects in the image using an adaptive thresholding method. Observing the histogram of the image color implies that a threshold can be determined to best differentiate between the background and the object distributions [42]. The threshold is computed by finding the histogram minimum (Figure 1) separating two peaks: the object and the background. However, the global minimum between the distributions is very hard to determine in most cases [43].

Currently, most optimal thresholding algorithms determine the threshold only in a one-dimensional space, for example in the RGB space, either R, or G, or B, or a linear combination of their values (e.g., grayscale transformation) [44]. In the transformation from three color dimensions into one, information is lost. In this research, a three-dimensional thresholding algorithm based on [19] was applied and evaluated also for additional color spaces (RGB, HSI, LAB, and NDI color spaces); a threshold is determined for each dimension in the color space.

There are two common adaptive thresholding algorithm concepts: (1) global thresholding, in which for each image, a different threshold is determined according to specific conditions for the entire image that is then transformed into a binary image; (2) local thresholding, in which the image is divided into sections and a different threshold is calculated for each section; the sections are then combined to a binary image. There are several methods that utilize dynamic local thresholding algorithms [45,46]. A common approach is to use multi-resolution windows that apply a bottom-up method, merging pixels while a criterion is met [45,46]. Another approach is the top down method, where the image is divided into subregions according to specific criteria. The top-down approach reduces execution speed and improves generalization [47] and was therefore used in this research.

The previously-developed algorithm by Vitzrabin et al., [19], which this research is based on, dynamically divides the image into several regions, each with approximately the same lighting conditions. The main contribution of the adaptive local 3D thresholding is a very high True Positive Rate (TPR) and a Low False Positive Rate (FPR) in the fruit detection task in an unstructured, highly-variable, and dynamic crop environment. Another contribution is the ability to change in real time the task objective to enable fast adaption to other crops, varieties, or operating conditions requiring small datasets and fast training. The algorithm’s adaptation to the desired ratio between TPR and FRP makes it specifically fit for robotic harvesting tasks for which it was originally designed; it contributes to a better success rate in robotic operations in which at first, FPR should be minimum (to reduce cycle times), and when approaching the grasping operation itself [19], TPR should be maximized (to increase grasping accuracy). This can be applicable towards other fruit detection tasks (e.g., same as above for spraying, thinning and in yield detection first maximizing TPR for deciding on harvesting timing and then minimizing TPR for accurate marketing estimation).

### 2.2. Color Spaces

Images can be represented by different color spaces (e.g., RGB, HSI, LAB, and NDI), each one emphasizing different color features [22]. RGB is the most common color space representing each pixel in the image in three color channels as acquired: red, green, and blue. HSI represents every color with three components: hue (H), saturation (S), and intensity (I), also known as HSV [37]. The LAB color space is an approximation of human vision [36] and presents for each pixel the L* (Lightness) from black to white, a* from green to red, and b* from blue to yellow. An additional color space commonly employed in the agriculture field [19] is the Normalized Difference Index (NDI) space. The NDI is used to differentiate between fruit and background [48] since it helps to overcome changes in illumination and shading due to its normalization technique [49]. Each dimension in the NDI space is the normalized difference index between two colors in the RGB space, resulting in three dimensions (Equation (1)). These operations are applied for all pixel locations in the image, creating a new image with this contrast index. These equations yield NDI values ranging between −1 and +1.

(1) $$NDI_{1}=\frac{R-G}{R+G};NDI_{2}=\frac{R-B}{R+B};NDI_{3}=\frac{B-G}{B+G}$$

## 3. Materials and Methods

### 3.1. Databases

**Figure 2:**
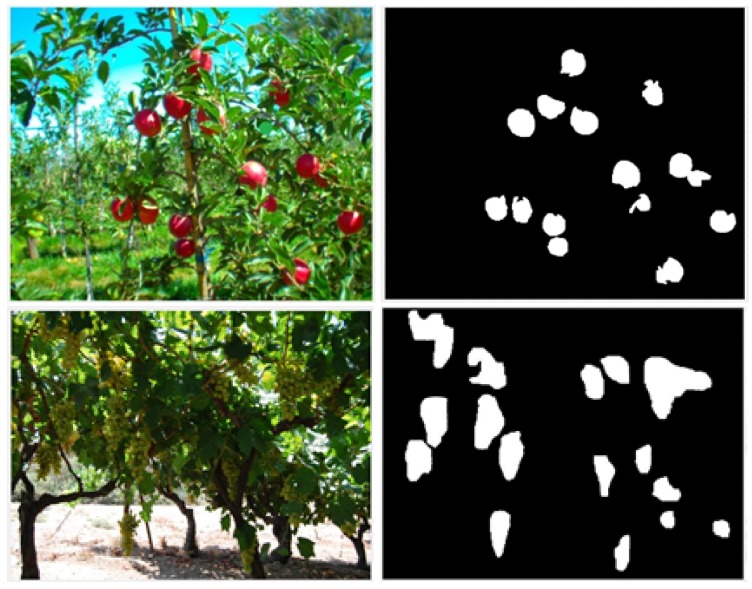
Apple (**top**) and grape (**bottom**) RGB image (**left**) and ground truth (**right**) examples.

**Figure 3:**
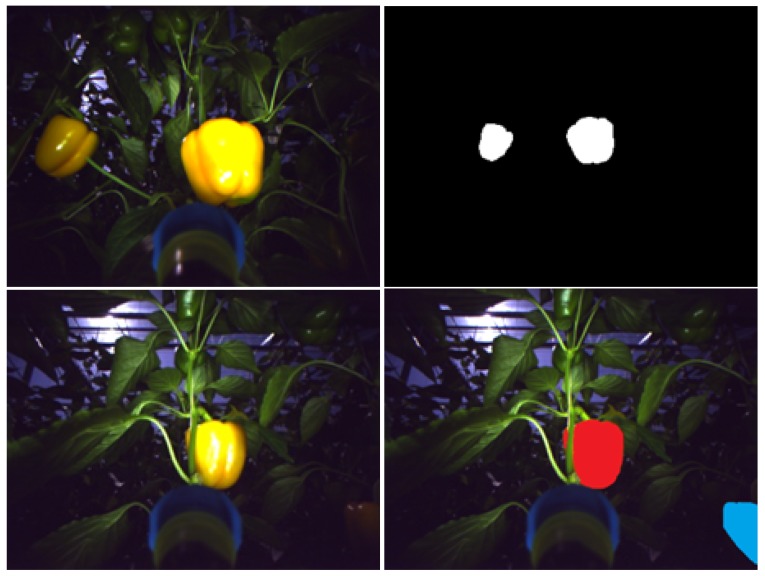
Peppers tagging example. **Top**: RGB image (**left**) and ground truth (**right**) example in high visibility. **Bottom**: RGB image (**left**) and labeled image (**Right**). “High-visibility peppers” marked in red and “low-visibility peppers” marked in blue.

The algorithm was evaluated on three databases representing three different fruit colors: red (apples), green (grapes), and yellow (peppers) and different types of fruits (high image and low image ratios) for two environmental settings (greenhouse and field) in different illumination conditions. Images were acquired with different cameras. Each image was processed by a human labeler who performed manual segmentation of the image into targets and background (Figure 2 and Figure 3) by visually analyzing the image and marking all the pixels considered as a fruit, in accordance with the common protocols used in the computer vision community [50].

#### 3.1.1. Apples

The orchard apples database included 113 “Royal Gala” apples in 9 images acquired from an orchard in Chile in March 2012 under natural growing conditions with a Prosilica GC2450C (Allied Vision Technologies GmbH, Stadtroda, Germany) camera with 1536 × 2048 resolution; the camera was attached to a pole. The images were captured in daylight; half of the images were acquired under direct sunlight, and half of the images were acquired in the shade.

#### 3.1.2. Grapes

The images were acquired in a commercial vineyard growing green grapes of the “superior” variety. An RGB camera (Microsoft NX-6000) with 600 × 800 resolution was manually driven, at mid-day, along a commercial vineyard in Lachish, Israel, during the summer season of 2011, one month before harvest time. The images were captured from five different growing rows. A set of 129 images was acquired and included 1078 grape clusters.

#### 3.1.3. Peppers

The dataset included 30 images of 73 yellow peppers acquired in a commercial greenhouse in Ijsselmuiden, Netherlands, using a 6 degree of freedom manipulator (Fanuc LR Mate 200iD/7L), equipped with an iDS Ui-5250RE RGB camera with 600 × 800 resolution. Two different datasets were created by marking the images twice. The first dataset included only peppers with high visibility (denoted as “high visibility peppers”; this was done for 10 images of 25 yellow peppers). In the second dataset, all peppers were marked including peppers in dark areas that were less visible in the image (denoted as “including low-visibility peppers”, done for all 30 images) (Figure 3).

#### 3.1.4. Performance Measures

Metrics included the TPR (True Positive Rate, also noted as hit), FPR (False Positive Rate, also noted as false alarms), and the F-score (the harmonic mean of precision and recall [51]. The TPR metric (Equation (2)) states the number of correctly-detected objects relative to the actual number of objects, while the FPR metric calculates the number of false objects detected relative to the actual number of objects (Equation (3)). The F-score (Equation (4)) balances between TPR and FPR equally.

(2) $$TPR=\frac{N_{TDF}}{N_{F}}$$

where $N_{TDF}$ is the number of pixels detected correctly as part of the fruit and $N_{F}$ is the actual number of pixels that represent the fruit.

(3) $$FPR=\frac{N_{FDF}}{N_{B}}$$

where $N_{FDF}$ is the number of pixels falsely classified as fruit and $N_{B}$ is the number of pixels that represent the background.

(4) $$F\left(TPR,FPR\right)=\frac{2*(TPR*(1-FPR))}{TPR+(1-FPR)}$$

### 3.2. Analyses

The following analyses were conducted for the three databases, apples, grapes, and peppers, using 70% of the data for training and 30% for testing [52]. This rate was chosen to make the algorithm performances more rigid since the number of images in each DB was relativity small. In addition, to ensure robust results, each split into training and testing was randomly performed 5 times, and all detection results reported are an average of the 5 test sets.

- Tuning parameters: Parameters were computed for each database with procedures defined in Section 4.3 and compared to previous predefined parameters
- Color spaces’ analyses: Algorithm performances were tested on all databases for four different color spaces: RGB, HSI, LAB, and NDI.
- Sensitivity analysis: Sensitivity analyses were conducted for all the databases and included:
  1. Noise: Noise was created by adding to each pixel in the RGB image a random number from the mean normal distribution for noise values up to 30%. The artificial noise represents the algorithms’ robustness toward other cameras with more noise, or when capturing images with different camera settings. Noise values of 5%, 10%, 20%, and 30%, were evaluated.
  2. Thresholds learned in train process: Thresholds were changed by ±5%, ±10%, and ±15% according to the threshold in each region.
  3. Stop condition: The selected STD value was changed by 5% and 10% to test the robustness of the algorithm to these parameters.
  4. Training vs. testing: The algorithm performances were evaluated while using different percentages of DB images for the training and testing processes.
- Morphological operation (erosion and dilation) contribution: Performances were tested for imaging with and without a morphological operations process.

## 4. Algorithm

### 4.1. Algorithm Flow

**Figure 4:**
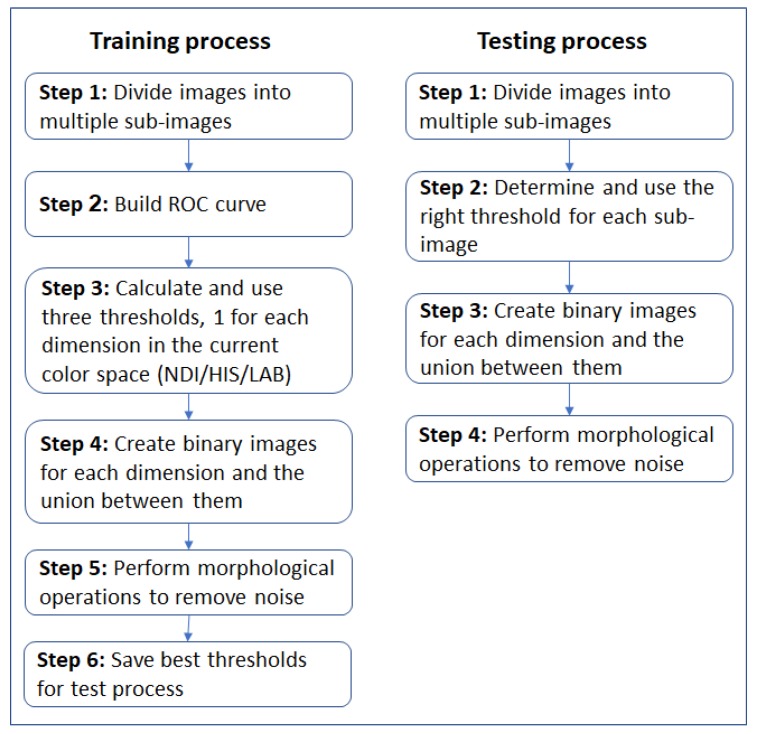
Algorithm flowchart.

**Figure 5:**
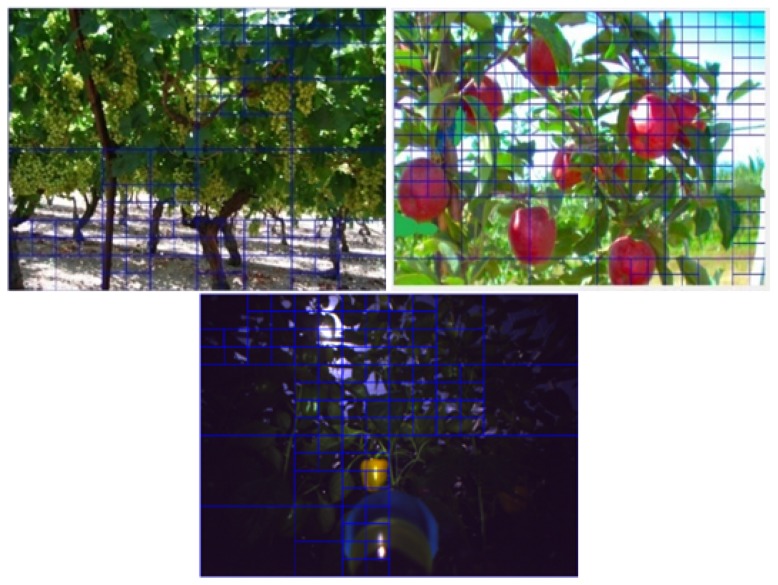
Image split into sub-images: visualization.

The overall flow of the algorithm is outlined in Figure 4, and it is as follows. The RGB images were the inputs for the training process. Some areas in the images contained more illumination than others, depending on the position of the light source and shading caused by leaves, branches, and the covering net when it existed. To overcome this issue, the algorithm divided each image into multiple sub-images, with approximately homogeneous illumination conditions (Figure 5). These sub-images were categorized into three illumination conditions: low, medium, and high. The illumination level was obtained by calculating the average on the grayscale sub-images. The grayscale image showed values between zero (completely dark) and 255 (completely white). In the previous algorithm [19], the sub-images were categorized into groups using levels selected empirically as 10, 70, and 130, corresponding to low-, medium-, and high-level images based on manual image analyses. The high value was set as 130 in order to filter overexposed areas in the images. In the current algorithm, a tuning parameter process (detailed in Section 4.3) was developed to determine these three values.

**Figure 6:**
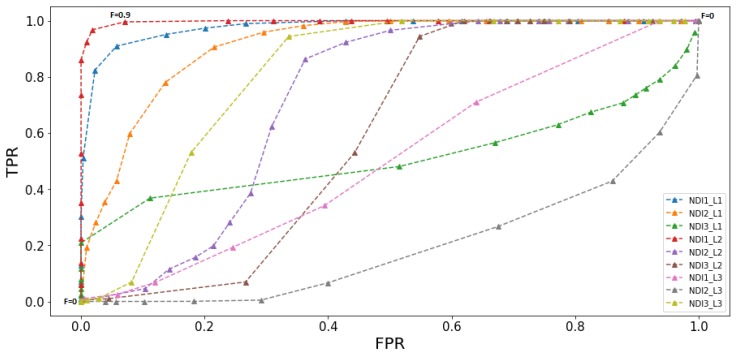
Nine ROC curves: 3 dimensions × 3 light levels $NDI_{i}-L_{j}$; *i* represents the color space dimension; *j* represents the illumination level.

The algorithm then created a 3D color space image (transformed the RGB image to NDI, HSI, and LAB space or used the RGB space directly). For each color dimension, a binary image (mask) was created, where each pixel that represents the fruit received a value of one and all other pixels received a value of zero. Finally, the algorithm created an ROC (Receiver Operator characteristics Curve) representing TPR as a function of FPR [53] including all nine thresholds learned from the training process. Figure 6 presents an example of nine ROC curves computed for three sub-images with different Light levels (L1, L2, L3) in the NDI color space. In this example, the sub-image with Light Level 2 (L2) in the first NDI dimension obtained the best performances (high TPR and low FPR).

In the test process, the algorithm received RGB images from the camera in real time, transformed the representation to the relevant color space (HSI/LAB/NDI), and created a binary image by applying the thresholds as follows: three thresholds, one for each dimension, were calculated from the nine thresholds learned by linear interpolation between two of the three illumination regions (low, medium, and high) selected as closest to the calculated illumination level for the specific sub-image from the grayscale image and using Equation (5).

(5) $$T=\frac{T(LL[i])*(currentLL-LL[i])(LL[i+1]-currentLL)}{LL[i+1]-LL[i]}$$

where LL is an array of the light level values for each group: LL = [low,medium,high]), and *i* is the light level index representing the group that is the closest to the current image light level from below.

For example, if the current light level is 40 and the thresholds in the train process for the low, medium, and high light levels were 10, 70, and 130, the threshold would be calculated in the following way (Equation (6)):

(6) $$T=\frac{T(10)(40-10)+T(70)(70-40)}{70-10}$$

**Figure 7:**
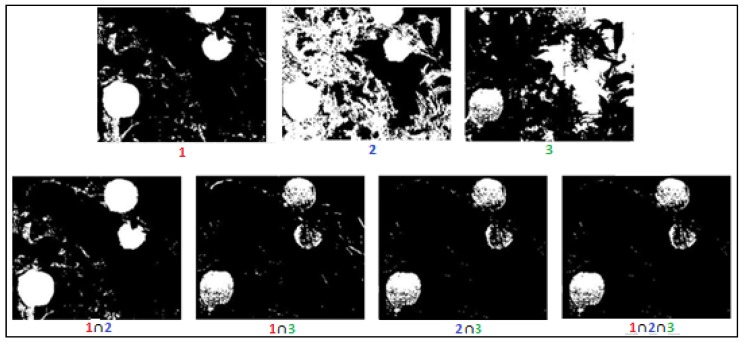
Use of dimension intersection to increase performance.

The end of the process results in binary images where white areas (pixels with a value of one) in the binary image represent the fruits and the black areas (pixels with a value of zero) represent the background (see Figure 2 and Figure 3). In total, the algorithm created 7 binary images, 3 images corresponding to the three color space dimensions and 4 binary images corresponding to the intersections between the first 3 binary images. For example, the intersection between Binary Images 1 and 2 resulted in a binary image 1∩2 that contained white pixels only, where the same pixels were white in both Images 1 and 2 (Figure 7).

### 4.2. Morphological Operations: Erosion and Dilation

**Figure 8:**
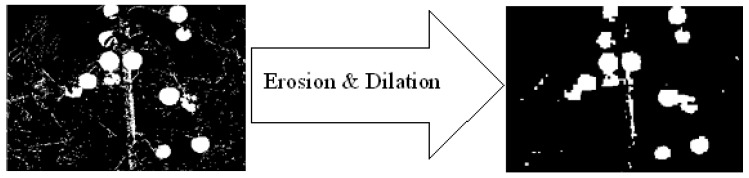
Morphological operation.

The algorithm result is a binary image with major fruit detected and small clusters of pixels that were wrongly classified as fruits (e.g., Figure 8). In addition, some fruits were split between several clusters (e.g., Figure 8). To overcome these problems, several morphological operations were performed based on previous research that indicated their contribution [19]: erosion followed by dilation with a neighborhood of 11 × 11-pixel squares. The square function was used since there was no pre-defined knowledge about the expected fruit orientation. To connect close clusters, the closing morphological operation was then applied by dilation followed by erosion implemented with a 5 × 5-pixel square neighborhood.

### 4.3. Parameter Tuning

**Figure 9:**
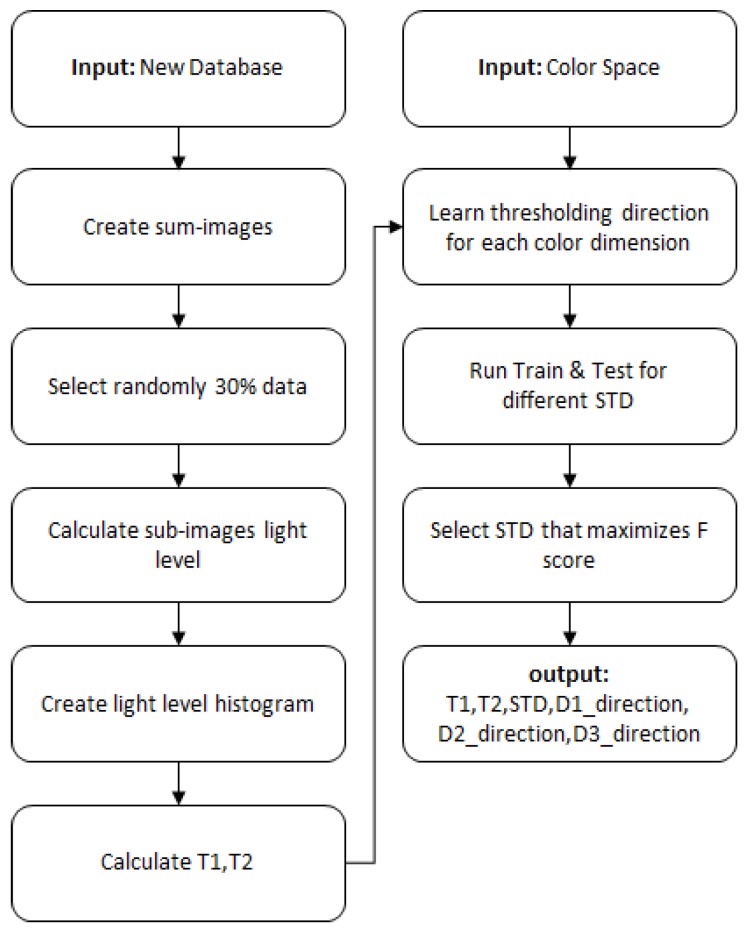
Parameter tuning process.

The algorithm used several parameters that influence the algorithm performances: T1, T2, STD, classification rule Direction (D1/D2). The following parameter tuning procedure (Figure 9) was developed and should be performed when exploring images from a new database or when exploring a new color space or new operating conditions (cameras, illumination). The parameters, as detailed below, are: light level thresholds, stop splitting condition, classification rule direction.

#### 4.3.1. Light Level Thresholds (T1, T2)

**Figure 10:**
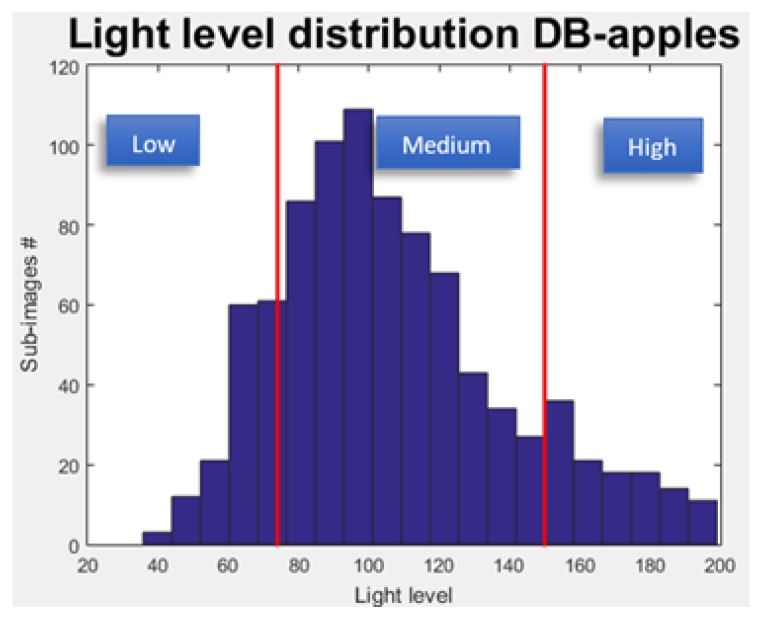
Sub-images level of light distribution.

The algorithm split the images into sub-images set to 1% pixels of the entire image. Then, the algorithm computed the light level of each sub-image by calculating the average pixels values of the grayscale sub-image. Finally, the algorithm grouped the sub-images into three light level categories (see Figure 10) using two thresholds as presented in Equation (7).

(7) $$i=\left\{\begin{array}{cc} Low & 0<x<T1\\ Medium & T1<x<T2\\ High & x>T2 \end{array}\right.$$

where *i* is the light level index, as detailed above in Equation (5).

Research was done to identify the PDF function of the data distributions of each database through a $\chi^{2}$ goodness of fit test. However, since these tests did not reveal significant results [54], the thresholds were selected as follows: T1 and T2 were chosen so that 15% of the data would be categorized as low, 15% as high, and 70% as medium.

Note that as described in the algorithm flow, the algorithm used a third threshold. Sub-images above that threshold were ignored in the training process since they were almost completely white.

#### 4.3.2. Stop Splitting Condition (STD)

The algorithm split an image into sub-images until the sub-image achieved a predefined Standard Deviation (STD) value. This approach assumes that a larger sub-image contains a higher STD value. To test this assumption, STD was calculated for different sizes of sub-images for the different databases. The stop condition value (STD minimum value) was determined by maximizing the F-score (Equation (4)).

#### 4.3.3. Classification Rule Direction (D1, D2, D3)

As detailed in the Introduction section, as part of the thresholding process, an intensity value was determined to differentiate between objects and background pixels. In order to execute the thresholding process, the algorithm must receive as input the classification rule direction (the algorithm must automatically determine if the intensity of the background distribution is higher or lower than the intensity of the object distribution in each color dimension).

This information was learned as part of the tuning procedure. A simple heuristic rule was used as follows based on the assumption that the images contained more background pixels than objects: (1) execute image>Threshold; (2) if the pixels categorized as background represent less than 70% of the image, reverse the thresholding direction images<Threshold.

## 5. Results and Discussion

### 5.1. Sub-Image Size vs. STD Value

**Figure 11:**
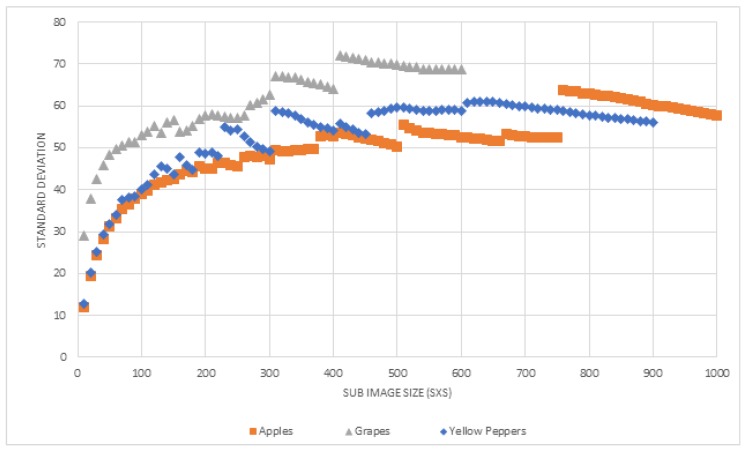
Sub image size vs. average STD.

For images with a size of 300 × 300 or lower, splitting an image into small sub-images (small S) decreases the average STD of the sub-images (in all three databases; Figure 11). Although a direct decrease in very large images is not noted, we still can conclude that splitting a large image to 300 × 300 or lower will decrease the average STD.

### 5.2. Tuning Process

This section presents the tuning procedure results, including thresholds derived to categorize the sub-images into light level groups, as well as the recursive stop condition that achieved the best result for each database.

#### 5.2.1. Light Level Distribution

**Figure 12:**
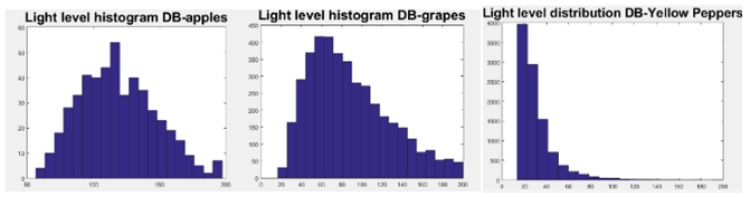
Light level distribution computed for each database.

The light level distribution was computed for each database (Figure 12) along with T1 and T2 (Table 2). The variation in the light distributions between the different databases are described in Table 3. The variance of light in the grape databases was significantly higher than in both the apple and the pepper databases, the pepper database being significantly darker and highly skewed. Therefore, for each database, the selected T1 and T2 were significantly different, implying the importance of the tuning procedure.

#### 5.2.2. Stop Splitting Condition

**Figure 13:**
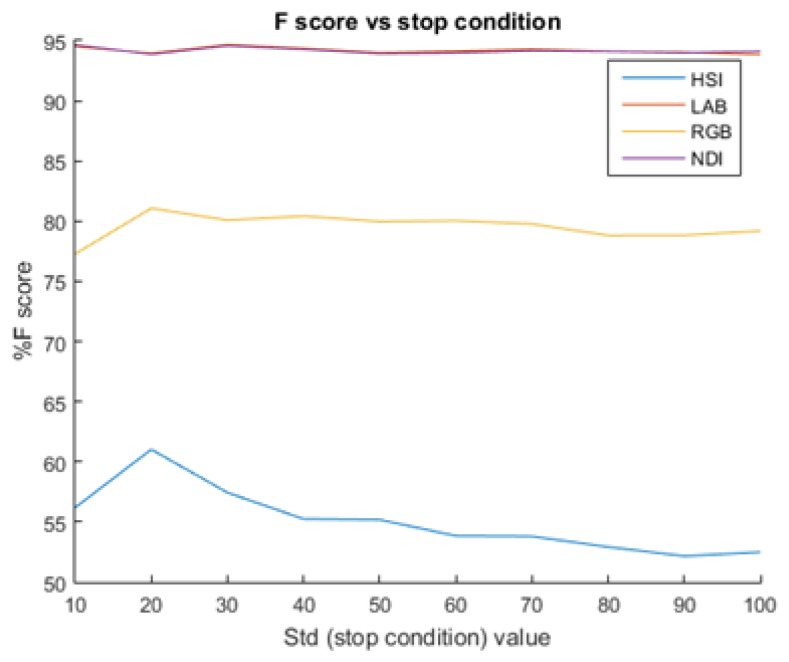
F-score vs. increasing STD value as the stop condition for the recursive function on the apple DB.

Using a low STD value as a stop condition increased the performance (Figure 13). This happens since smaller sub-images contain less illumination differences. However, small STD values can create also too small sub-images, which may not contain fruit and background pixels in the same frame. In these cases, the algorithm cannot learn a threshold that could differ between them. Additionally, results revealed that when using high STD values, the performances remained constant. This happens since beyond a certain value, the algorithm did not split the image even once.

As part of the parameter tuning process, the STD value was selected by testing the performances of a range of STD [0, 100]. For each STD value, the algorithm ran five iterations where it randomly selected P% of the images, from the selected images; it used 70% for training and 30% testing. The final selected STD values are presented in Table 4 for each database and color space (using P = 30% and 50%).

#### 5.2.3. Classification Rule Direction

As shown in Table 5, the direction of the classification rule in the thresholding process can be different for each color dimension; therefore, this must be learned as part of the tuning procedure.

### 5.3. Color Space Analyses

In this section, algorithm performance results are presented for each color space followed by a table representing the best color space performances including the performances for all color space dimensions’ combinations.

#### 5.3.1. Apples

**Figure 14:**
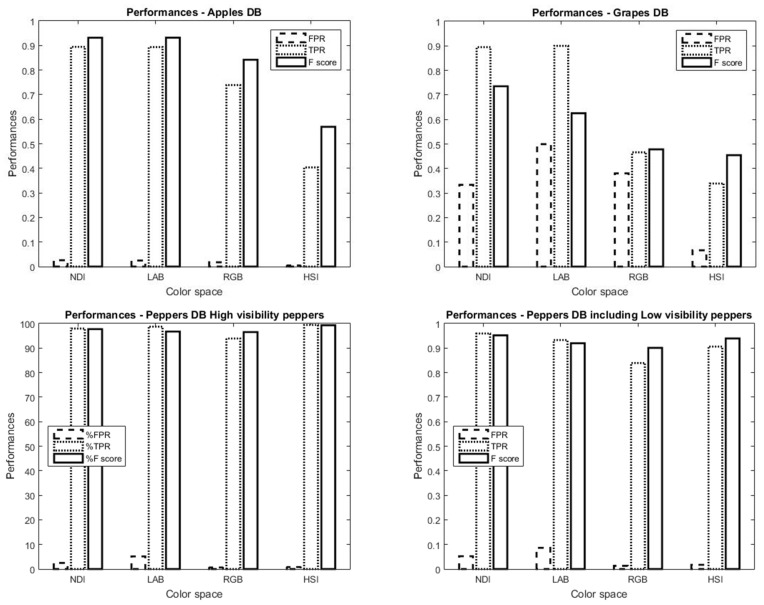
Color space performances for (**top**) apples (**left**), grapes (**right**), peppers (**bottom**) with high visibility (**left**), and peppers with low visibility (**right**).

Results (Figure 14) revealed that NDI and LAB color spaces resulted in similar best performances. In Table 5, the preferences for each dimension in the NDI color space and the performances when using the intersection between them is shown. The NDI first dimension (see Equation (1)) represents the difference between the red and green colors in the image. The objects in this database were red apples, and most of the background was green leaves; therefore, as expected, the first NDI obtained the best F of 93.17%. In the LAB color space, results (Table 5) revealed that the second dimension (A) yielded the best F-score of 93.19.

#### 5.3.2. Grapes

The NDI color space obtained the best result for grapes (Figure 14) with an F-score of 73.52%. The second-best color space was the LAB with an F-score of 62.54%. The best NDI results were obtained using the second dimension (Table 5).

#### 5.3.3. Peppers

High visibility: Figure 14 indicates that the HSI color space obtained the best results with relatively low FPR (0.81%) and very high TPR (99.43%), resulting in a high F-score (99.31%). The second-best color space was NDI with FPR = 2.48% and TPR = 97.96% (F = 97.72%). The best HSI result was obtained using the combination of the first and the second dimensions (Table 5).

Including low visibility: Figure 14 indicates that the NDI color space obtained the best results with relatively low FPR (5.24%) and very high TPR (95.93) resulting in a high F-score (95.19%). Although for the “high visibility” peppers HSI obtained the best performances, when trying to detect peppers in dark areas that were less visible, NDI showed better results. The best NDI result was obtained using the intersection between the first and the second dimensions (Table 5).

### 5.4. Sensitivity Analysis

#### 5.4.1. Noise

**Figure 15:**
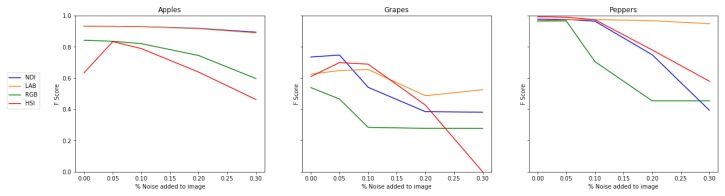
Sensitivity analysis: adding noise to the image.

Analysis showed that the algorithm was robust to noise in the image up to 15% in the apple and pepper databases (Figure 15). The grape images were more sensitive to noise, and performance dropped when noise values of 5% were added. Although better F-score values were obtained for NDI and HSI for grapes and peppers, we can see that the LAB color space yielded more robust performance when adding noise to the images.

#### 5.4.2. Thresholds Learned in the Training Process

As expected, TPR decreased when the threshold values changed. The algorithm was relatively robust to the change in the thresholds for apples and peppers. Performance in the grape images was more sensitive to threshold changes, and yielded a significant decrease in TPR when increasing the threshold value (Table 6).

#### 5.4.3. Stop Condition

**Figure 16:**
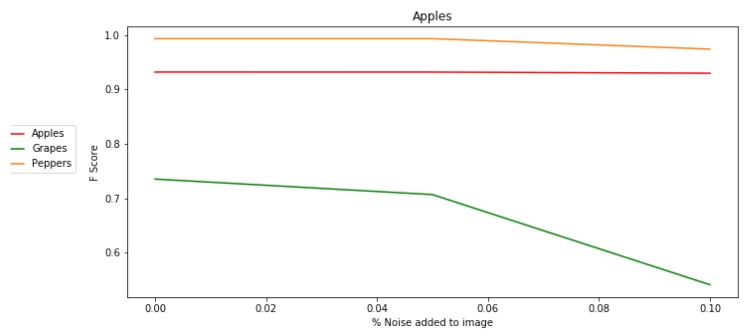
Sensitivity analysis: adding noise to STD stop condition.

The algorithm showed more robustness to apple and pepper images than grapes (Figure 16).

#### 5.4.4. Training/Testing

The expectation was that more training images would lead to better performance until over fitting was accommodated. There was a clear increase in TPR; however, FPR increased as well at 80% and 90% training (Table 7).

The tuning process resulted (Table 8) in increased performances for both the grape and pepper databases with a 6.22% and 0.84% increase, respectively. The results for the apple database were similar with only a 0.1% increase, as expected (since this was similar to the database from which the previous parameters were derived).

### 5.5. Morphological Operations

**Figure 17:**
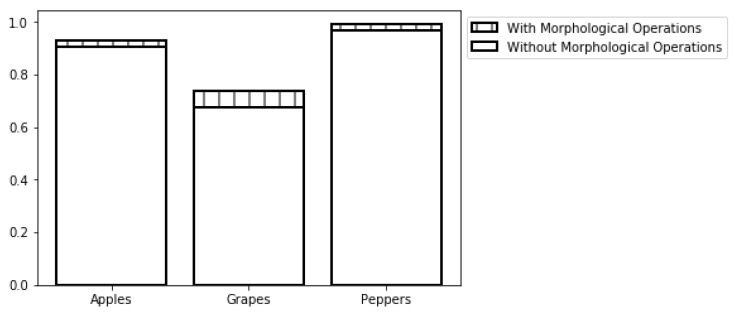
Sensitivity analysis: adding noise to the STD stop condition.

The morphological operations process increased the F-score by 2.85%, 8.59%, and 2.71% for the apple, grape, and pepper databases respectively, (Figure 17).

## 6. Conclusions and Future Work

The algorithm successfully detected apples and peppers (Table 1) in variable lighting conditions resulting in an F-score of 93.17% and 99.31%, respectively, which is one of the best detection rates achieved to date in fruit detection to the best of our knowledge. The average F-score across all datasets was 88.8 (Table 1). Previous research achieved the lowest F-score (65) with the method of [11] for red and green pepper plants, while oranges obtained the highest F-score (96.4) with that of [24]. Previous reported results (Table 1) revealed a 91.5 and 92.6 F-score for peppers in [19,34], respectively, versus our method, which resulted in an F-score of 99.43. For apple images, our method obtained similar F-score performances as in previous work (~93), even though the dataset was much smaller (64 vs. 9 images).

The high F-score was mostly due to low FPR values (except for grapes). In addition, our method achieved high performances using a relatively small dataset.

The algorithm resulted in less impressive results in the grape database of 73.52% due to the difficulties in differentiating between green fruits and green background (leaves). In this case, additional features (e.g., morphological operations fitted for grapes; see [28]) should be used to increase performance. However, this requires the development of specially-tailored features. It is important to note that these results cannot be compared to the weed detection results presented in Table 1, since the background of the green obkects was the ground on which it grew and not the green leaves. Different color spaces yielded the best results for each fruit variety, implying that the color space must be analyzed and fitted to the specific fruit. The LAB color space was more robust to noise in images and hence should be used when images are of low quality. The algorithm was robust to changes in the threshold learned by the training process and to noise effects in images. Morphological operations such as erosion and dilation can improve performance in agriculture images and hence should be utilized. The tuning process developed in this paper enabled the previous algorithm [30] to adapt automatically to changing conditions/objectives (i.e., to detect other fruit with different colors and other outdoor conditions) and, hence, should be used for improved target detection in highly-variable illumination conditions. Finally, this work has presented the feasibility of color-based algorithms solving the challenges that advanced machine learning algorithms face such as small training sets (small number of images and/or small number of fruits per image). This work has shown that for challenging color conditions (e.g., green on green for grapes), additional features should be considered for improved fruit detection. 

## Figures and Tables

**Table 1:** Summary of previously-published results.

Paper	Crop	Dataset Size	Detection Level	Algorithm Type	FPR %	TPR %	F %	A %	P %	R %
Arad et al., 2019 [20]	Peppers	156 img	PX	AD	NA	NA	NA	NA	65(95)	94(95)
			W	DL					84	NA
Ostovar et al., 2018 [34]	Peppers	170 img	PX	AD	NA	NA	NA	91.5	NA	NA
Chen et al., 2017 [24]	Apples	1749 (21 img)	PX	DL	5.1	95.7	95.3 *	NA	NA	NA
	Oranges	7200 (71 img)			3.3	96.1	96.4 *			
McCool et al., 2017 [26]	Weed	Pre-train:$10^{6}$ img tuning and testing: 60 img	PX	D-CNN	NA	NA	NA	93.9	NA	NA
Milioto et al., 2017 [27]	Weed	5696 (867 img) 26,163 (1102 img)	PX	CNN	NA	NA	NA	96.8 99.7	97.3 96.1	98.1 96.3
Sa et al., 2016 [23]	Sweet pepper	122 img	W	DL	NA	NA	82.8	NA	NA	NA
	Rock melon	135 img					84.8			
	Apple	64 img					93.8			
	Avocado	54 img					93.2			
	Mango	170 img					94.2			
	Orange	57 img					91.5			
Vitzrabin et al., 2016 [19]	Sweet pepper	479 (221 img)	PX	AD	4.6	90.0	92.6 *	NA	NA	NA
Song et al., 2014 [11]	Pepper plants	1056 img	W	NB+SVM	NA	NA	65	NA	NA	NA
Nuske et al., 2011 [6]	Grapes	2973 img	PX	K-NN	NA	NA	NA	NA	63.7	98
Berenstein et al., 2010 [28]	Grapes	100 img	PX	Morphological	NA	NA	NA	90	NA	NA
Zheng et al., 2009 [37]	Vegetation	20 img 80 img	PX	Mean-Shift	NA	NA	NA	95.4 95.9	NA	NA
Our results	Sweet pepper	73 (30 img)	PX	AD	0.81	99.43	99.31	NA	NA	NA
	Apples	113 (9 img)			2.59	89.45	93.17			
	Grapes	1078 (129 img)			33.35	89.48	73.52			

DL = Deep Learning; PX = Pixels’ segmentation; AD = Adaptive threshold; NB = Naive Bias; W = Window detection; F = F-score; A = Accuracy; P = Precision; R = Recall; * Calculated F-score based on reported TPR and FPR according to Equation (4).

**Table 2:** T1 and T2 values determined for each database.

Measure\DB	Apples	Grapes	Peppers
**T1**	84	49	18
**T2**	140	130	47

**Table 3:** Descriptive statistics of the different light distributions.

DB\Measure	Mean	Std	Skewness	Kurtosis	Median
**Apples**	118.46	28.04	0.4	−0.17	116.31
**Grapes**	88	37.9	0.68	−0.13	81.06
**Peppers**	32.09	18.92	3.16	15.36	26.93

**Table 4:** STD value chosen for each database and color space. D, Direction.

DB		Apples				Grapes				Peppers			
Color space		HSI	LAB	NDI	RGB	HSI	LAB	NDI	RGB	HSI	LAB	NDI	RGB
STD (P = 30%)		20	30	10	20	10	20	60	20	100	10	10	10
STD (P = 50%)		20	10	10	30	20	20	70	20	100	20	10	10
Classification rule direction	D1	>	<	>	>	<	>	>	>	<	>	>	>
	D2	>	>	>	<	>	<	>	>	>	>	>	>
	D3	>	>	>	<	<	>	<	<	>	>	<	<

**Table 5:** Performances of each color space for each dimension and intersection for all datasets.

			Dimension						
DB	Color Space	Measure	1	2	3	1∩2	1∩3	2∩3	1∩2∩3
**Apples**	**NDI**	**% FPR**	**2.59**	40.91	31.38	1.64	1.32	2.48	0.48
		**% TPR**	**89.45**	83.53	68.39	78.52	64.82	54.65	54.1
		**% F**	**93.17**	67.85	67.8	86.75	77.6	69.67	69.69
	**LAB**	**% FPR**	33.58	**2.45**	77.08	1.78	28.55	1.55	1.07
		**% TPR**	61.26	**89.34**	85.26	56.59	52.95	76.27	48.8
		**% F**	56.79	**93.19**	35.85	69.02	54.61	85.37	62.58
**Grapes**	**NDI**	**% FPR**	35.86	33.35	52.9	4.86	5.5	**32.35**	4.09
		**% TPR**	44.52	89.48	89.99	38.53	37.27	**87.5**	36.7
		**% F**	47.19	73.52	58.05	50.12	48.93	**73.2**	48.65
**Peppers** **High** **Visibility**	**HSI**	**% FPR**	18.27	1.52	2.16	**0.81**	0.64	0.17	0.14
		**% TPR**	99.96	99.43	86.42	**99.43**	86.41	86.23	86.23
		**% F**	89.77	98.95	91.41	**99.31**	92.12	92.24	92.25
**Peppers** **Inc. Low** **Visibility**	**NDI**	**% FPR**	66.57	**5.24**	9.23	1.42	1.24	4.57	0.99
		**% TPR**	85.61	**95.93**	92.49	82.2	78.64	92.33	78.61
		**% F**	46.96	**95.19**	91.24	88.91	86.59	93.51	86.67

**Table 6:** Threshold values changed by ±5%, ±10%, and ±15% according to the threshold in each region.

		Changes in Threshold						
DB	Measure	−15%	−10%	−5%	0%	5%	10%	15%
**Apples**	**% FPR**	3.58	3.43	3.3	2.59	3.06	2.93	2.81
	**% TPR**	91.47	91.28	91.07	89.45	90.75	90.57	90.44
**Grapes**	**% FPR**	21.59	18.4	15.53	33.35	11.00	9.23	7.72
	**% TPR**	78.02	72.63	66.42	89.48	50.99	43.63	36.24
**Peppers**	**% FPR**	0.98	0.91	0.86	0.81	0.78	0.7	0.65
	**% TPR**	99.25	99.22	99.2	99.43	99.12	99.07	99.04

**Table 7:** Performances vs. different % images database as the training set.

		Training %: For a Dataset Size of 129 Images								
DB	Measure	10	20	30	40	50	60	70	80	90
**Grapes**	% FPR	32.81	37.08	28.54	36.16	31.83	29.1	29.63	40.51	40.8
	% TPR	88.79	89.62	87.01	88.44	87.58	82.55	87.14	94.53	95.85
	% F	73.35	70.2	75.19	69.41	72.49	70.24	73.92	72.55	72.54

**Table 8:** Parameter tuning contribution to algorithm performances.

DB	Measure	Performances Using Previous Params	Performances Using Tuning Process
**Apples**	**% FPR**	2.53	2.59
	**% TPR**	89.23	89.45
	**% F**	93.08	93.17
**Grapes**	**% FPR**	18.63	33.35
	**% TPR**	63.7	89.48
	**% F**	67.3	73.52
**Peppers**	**% FPR**	1	0.81
	**% TPR**	97.97	99.43
	**% F**	98.47	99.31
